# Defining sarcopenia and myosteatosis: the necessity for consensus on a technical standard and standardized cut‐off values

**DOI:** 10.1002/jcsm.12961

**Published:** 2022-02-26

**Authors:** Lisa B. Westenberg, Marcel Zorgdrager, Alain R. Viddeleer, Robert A. Pol

**Affiliations:** ^1^ Department of Surgery, Division of Transplant Surgery University Medical Center Groningen, University of Groningen Groningen The Netherlands; ^2^ Department of Radiology, Medical Imaging Center University Medical Center Groningen, University of Groningen Groningen The Netherlands

With great interest we read the paper by Morel *et al*.^1^ in the *Journal of Cachexia, Sarcopenia and Muscle*. The authors presented an interesting study on the impact of low skeletal muscle mass index (as a surrogate marker for sarcopenia) and low muscle density (as a surrogate marker for myosteatosis) on patient survival after kidney transplantation. They performed measurements on cross‐sectional computed tomography (CT) scans taken at the level of the third lumbar vertebra. We agree with the authors that the analysis of CT scans has the advantage of being able to not only provide information about muscle quantity but also its quality, contributing to a more accurate assessment of body composition (components) and related health risks.

We feel there is a need to emphasize one important aspect of the discussion. To date, there is still no consensus on standardized CT‐derived cut‐off values for low skeletal muscle mass and myosteatosis, which impairs accurate data analysis, interpretation, and subsequent translation to clinical practice. Although some studies have presented cut‐off values for low muscle mass^2^, ^3^, ^4^, ^5^, ^6^, ^7^ and low muscle density,^8^, ^9^ discrepancies exist between these cut‐off values due to differences in characteristics of the study population or the techniques used to assess quantity and quality of muscle mass, forcing researchers and clinicians to continue determining cut‐off values specific to their study population. Morel *et al*. made use of age‐specific and sex‐specific normality thresholds of 130 healthy subjects and used a standardized procedure for their CT examinations. The mean skeletal muscle index of healthy subjects found in their study slightly differs from that found in our cohort of almost 1000 living kidney donors (mean skeletal muscle index (in cm^2^ /m^2^) is 53.1 ± 7.3 in men and 42.0 ± 4.8 in women in our cohort *versus* 54.8 ± 7.9 in men and 41.7 ± 5.5 in women in the cohort of Morel *et al*.), and mean muscle density is lower in comparison to our cohort for both men and women (mean muscle density (in Hounsfield Units) is 49.3 ± 7.4 in men and 47.6 ± 7.9 in women in our cohort *versus* 43.8 ± 7.7 in men and 37.0 ± 8.2 in women in the cohort of Morel *et al*.), possibly due to differences in for example slice thickness. Several studies report an influence of technical parameters on muscle density, in which a higher slice thickness results in a lower muscle density and intravenous contrast and low tube current are associated with an increased muscle density.^10^, ^11^ The impact of variances in these technical parameters on skeletal muscle area measurements seems clinically less important, but requires further research.^10^, ^11^, ^12^ Due to increased attention in clinical practice and research for muscle quality, and the effect on various outcome measures, we need to develop a technical standard and formulate clear cut‐off values. Additionally, technical parameters should be reported in studies using CT for body composition analysis.

Healthy individuals undergoing CT as part of standard care, such as living kidney donors, provide an excellent opportunity to assess reference and cut‐off values of sarcopenia and myosteatosis, and future research and discussion should focus on establishing standardized procedures and achieve a proper validation.

## Conflict of interest

Lisa Westenberg, Marcel Zorgdrager, Alain Viddeleer, and Robert Pol declare that they have no conflict of interest.

## References

[1] Morel A , Ouamri Y , Canoui‐Poitrine F , Mule S , Champy CM , Ingels A , et al. Myosteatosis as an independent risk factor for mortality after kidney allograft transplantation: a retrospective cohort study. J Cachexia Sarcopenia Muscle 2022. 13:386–396. 34738343 10.1002/jcsm.12853 PMC8818595

[2] Derstine BA , Holcombe SA , Ross BE , Wang NC , Su GL , Wang SC . Skeletal muscle cutoff values for sarcopenia diagnosis using T10 to L5 measurements in a healthy US population. Sci Rep 2018;8:11369. 30054580 10.1038/s41598-018-29825-5 PMC6063941

[3] van der Werf A , Langius JAE , de van der Schueren MAE , Nurmohamed SA , van der Pant K , Blauwhoff‐Buskermolen S , et al. Percentiles for skeletal muscle index, area and radiation attenuation based on computed tomography imaging in a healthy Caucasian population. Eur J Clin Nutr 2018;72:288–296. 29242526 10.1038/s41430-017-0034-5 PMC5842880

[4] Ufuk F , Herek D . Reference skeletal muscle mass values at L3 vertebrae level based on computed tomography in healthy Turkish adults. Int J Gerontol 2019;13:221–225.

[5] Kim JS , Kim WY , Park HK , Kim MC , Jung W , Ko BS . Simple age specific cutoff value for sarcopenia evaluated by computed tomography. Ann Nutr Metab 2017;71:157–163. 28881338 10.1159/000480407

[6] Hamaguchi Y , Kaido T , Okumura S , Kobayashi A , Hammad A , Tamai Y , et al. Proposal for new diagnostic criteria for low skeletal muscle mass based on computed tomography imaging in Asian adults. Nutrition 2016;32:1200–1205. 27292773 10.1016/j.nut.2016.04.003

[7] van Vugt JLA , van Putten Y , van der Kall IM , Buettner S , D'Ancona FCH , Dekker HM , et al. Estimated skeletal muscle mass and density values measured on computed tomography examinations in over 1000 living kidney donors. Eur J Clin Nutr 2019;73:879–886. 30143785 10.1038/s41430-018-0287-7

[8] Czigany Z , Kramp W , Lurje I , Miller H , Bednarsch J , Lang SA , et al. The role of recipient myosteatosis in graft and patient survival after deceased donor liver transplantation. J Cachexia Sarcopenia Muscle 2021;12:358–367. 33525056 10.1002/jcsm.12669 PMC8061365

[9] Martin L , Birdsell L , Macdonald N , Reiman T , Clandinin MT , McCargar LJ , et al. Cancer cachexia in the age of obesity: skeletal muscle depletion is a powerful prognostic factor, independent of body mass index. J Clin Oncol 2013;31:1539–1547. 23530101 10.1200/JCO.2012.45.2722

[10] Fuchs G , Chretien YR , Mario J , Do S , Eikermann M , Liu B , et al. Quantifying the effect of slice thickness, intravenous contrast and tube current on muscle segmentation: implications for body composition analysis. Eur Radiol 2018;28:2455–2463. 29318425 10.1007/s00330-017-5191-3

[11] van Vugt JLA , van den Coebergh Braak RRJ , Schippers HJW , Veen KM , Levolger S , de Bruin RWF , et al. Contrast‐enhancement influences skeletal muscle density, but not skeletal muscle mass, measurements on computed tomography. Clin Nutr 2018;37:1707–1714. 28743427 10.1016/j.clnu.2017.07.007

[12] Derstine BA , Holcombe SA , Goulson RL , Ross BE , Wang NC , Sullivan JA , et al. Quantifying sarcopenia reference values using lumbar and thoracic muscle areas in a healthy population. J Nutr Health Aging 2017;21:180–185.10.1007/s12603-017-0983-329300439

[13] von Haehling S , Coats AJS , Anker SD . Ethical guidelines for publishing in the Journal of Cachexia, Sarcopenia and Muscle: update 2021. J Cachexia Sarcopenia Muscle 2021;12:2259–2261. 34904399 10.1002/jcsm.12899 PMC8718061

